# A Path Integral Molecular Dynamics Simulation of a Harpoon-Type Redox Reaction in a Helium Nanodroplet

**DOI:** 10.3390/molecules26195783

**Published:** 2021-09-24

**Authors:** Alvaro Castillo-García, Andreas W. Hauser, María Pilar de Lara-Castells, Pablo Villarreal

**Affiliations:** 1 Instituto de Física Fundamental, IFF-CSIC, Serrano 123, ES-28006 Madrid, Spain; garca926@gmail.com; 2 Institute of Experimental Physics, Graz University of Technology, Petersgasse 16, 8010 Graz, Austria; andreas.w.hauser@gmail.com

**Keywords:** helium nanodroplets, path integral molecular dynamics simulations, redox reaction, charge transfer process, superfluid helium, ab initio calculations, non-adiabatic couplings, harpoon mechanism

## Abstract

We present path integral molecular dynamics (PIMD) calculations of an electron transfer from a heliophobic Cs${}_{2}$ dimer in its (${}^{3}\Sigma_{u}$) state, located on the surface of a He droplet, to a heliophilic, fully immersed C${}_{60}$ molecule. Supported by electron ionization mass spectroscopy measurements (Renzler et al., *J. Chem. Phys.* **2016**, *145*, 181101), this spatially quenched reaction was characterized as a harpoon-type or long-range electron transfer in a previous high-level *ab initio* study (de Lara-Castells et al., *J. Phys. Chem. Lett.* **2017**, *8*, 4284). To go beyond the static approach, classical and quantum PIMD simulations are performed at 2 K, slightly below the critical temperature for helium superfluidity (2.172 K). Calculations are executed in the NVT ensemble as well as the NVE ensemble to provide insights into real-time dynamics. A droplet size of 2090 atoms is assumed to study the impact of spatial hindrance on reactivity. By changing the number of beads in the PIMD simulations, the impact of quantization can be studied in greater detail and without an implicit assumption of superfluidity. We find that the reaction probability increases with higher levels of quantization. Our findings confirm earlier, static predictions of a rotational motion of the Cs${}_{2}$ dimer upon reacting with the fullerene, involving a substantial displacement of helium. However, it also raises the new question of whether the interacting species are driven out-of-equilibrium after impurity uptake, since reactivity is strongly quenched if a full thermal equilibration is assumed. More generally, our work points towards a novel mechanism for long-range electron transfer through an interplay between nuclear quantum delocalization within the confining medium and delocalized electronic dispersion forces acting on the two reactants.

## 1. Introduction

Nanodroplets of ${}^{4}$He and/or ${}^{3}$He of various sizes provide ideal conditions for the spectroscopy of unstable or highly reactive impurities due to the inert and extremely cold environment they offer. Acting as storage containers or “quantum matrix”, He Nanodroplets (HNDs) were used to study a broad variety of molecular species in neutral or anionic states via electronic or infrared spectroscopy [1,2,3,4,5,6,7]. Experiments involving HNDs in which the species to study are captured by a beam of helium droplets in a low-pressure pick-up cell became nowadays usually combined with photoionization [8] and electron ionization mass spectrometry [9,10,11] techniques. A summary of the applications of helium HNDs in the study of clusters and complexes formed through collisions inside helium nanodroplets, as well as a detailed overview of the many studies of ions, both positive and negative, that were carried out in HNDs can be found in a recent review [12].

Qualitatively, the solvation process of atomic or molecular impurities in helium droplets is determined by the relative strengths of the He-impurity and He-He interactions. If the former dominates over the latter, the impurity is likely to become fully immersed, but it tends to stay outside otherwise. Attraction between any two helium atoms is indeed very weak (only ∼8 cm${}^{-1}$), and thus, is weaker than most of the He-dopant interactions. However, species such as alkali metal atoms or clusters thereof are known to remain on the droplet surface due to their particularly diffusive electron densities [13]. Their dimers behave in the same way and were widely investigated by the groups of Ernst and Stienkemeier: predictions for the observation of KRb and Rb${}_{2}$ spectra under cold conditions were made [14], and HNDs experiments on Na${}_{2}$ [15] or Rb${}_{2}$ were performed [16]. The general conclusion is that the diatomic is located in a dimple on the surface of the helium nanodroplet, which causes slight modifications in the spectroscopic properties of the impurity [17]. Indeed, alkali dimers in their lowest triplet state were formed on the surface of cold helium droplets [18], as it is expected due to the very weak high-spin alkali dimers and helium atoms interaction, and since then, their dynamics were extensively studied (see Refs. [19,20,21]). As observed earlier in experiments with alkali doped clusters [22], the alkali metal atoms remain on the helium surface, where they can *skate*, forming cold molecules through collisions [18,23]. In general, such collisions lead preferentially to high spin (triplet) state alkali dimers [16,18], as the binding energy that is released during the collision is larger for the low spin (singlet) state for which the subsequent evaporation of the droplet could be significant. In fact, singlet states with a stronger bond may cause either the desorption of the alkali molecule or the complete evaporation of small helium droplets, and thus, triplet-state alkali dimers were selectively produced [3]. The collisional formation of alkali dimers was addressed in the presence of just one helium atom to analyze the feasibility of the *skating* mechanism mentioned above, with the conclusion that at ultra-low collisional energies, the process is highly efficient, and the alkali dimer is formed at high-vibrational excitations [24]. By considering two impurities of different behavior in helium, experimental results using electron ionization mass spectrometry [25] show that heliophobic alkalis can be induced to submerge into liquid helium when a heliophilic, highly polarizable, cosolute as C${}_{60}$ is added to a helium nanodroplet. Indeed, it is concluded that there is a high probability for the formation of a Cs${}_{2}$-C${}_{60}$ complex inside the droplet, as it was also proposed that a direct harpoon-type electron transfer mechanism is responsible for the complex formation.With the helium medium being an electrical insulator unless ionized, the question of the physical mechanism making possible electron migration between donor and acceptor species at long-range is truly fundamental.

From the theoretical point of view, several methodologies were used to deal with impurities and their reactions in HNDs. Among them, density functional theory applied to bosonic helium droplets, which implicitly incorporates the helium superfluidity, and where the dynamics are real-time [26]. The capture of one Ar atom [27] or up to six Ar atoms [28], and the formation of Ne dimers [29] or Ne-Ne adducts [30] in HNDs were recently studied using this methodology. In turn, and including explicitly the temperature, path integral Monte Carlo (PIMC) techniques [31,32] constitute an efficient alternative to study energies and equilibrium structures of different species attached to bosonic helium droplets, see for instance the studies on alkali dimers, Cs${}_{2}$ [33] and Rb${}_{2}$ [34,35,36] or on coronene [37,38] and on lithium ions [39]. Note that all of these studies were restricted to sizes of a few hundred of helium atoms. However, as will be detailed further below, the inclusion of thousands of He atoms is necessary to capture all relevant effects of molecular motion. Therefore, we decided to perform path-integral molecular dynamics (PIMD) simulations [40] in the NVT ensemble, similar to a recent study on the formation of rubidium dimers on the surface of helium droplets [41]. The dynamics generated by PIMD (or PIMC) are not real-time, and issues arise concerning ergodicity, but the method can provide crucial information on the actual reaction mechanism, which is not accessible by any static approach. In addition, by turning off the thermostat, real-time dynamics can be attained by launching an extensive batch of trajectories to overcome ergodicity issues. Moreover, the present study involves rather large, heavy impurities, and despite the quantum character of the helium environment, it may be expected that the impurities follow an essentially classical behavior. For the sake of completeness, we also carry out illustrative simulations in the NVE ensemble.

A fundamental question of low-temperature physics concerns the understanding of frictionless motion of impurities in a superfluid environment at atomic scale. Studies of chemical reactions, including nuclear as well as electronic reconfiguration, two processes on vastly different time scales, are particularly interesting in this context. In this work, we chose the formation of ionic bonds as a suitable reaction to investigate the impact of friction and the pick-up of two reactants with significantly different solvation behavior; initially, the heliophilic reactant becomes fully immersed, while the heliophobic reactant remains on the surface of the He droplet. This way, a spatial hindrance for this electron-transfer reaction is introduced to the system. For the reaction between the heliophobic Cs or Cs${}_{2}$ species with the heliophilic C${}_{60}$ molecule in a helium droplet, previous simulations showed clear evidence for the occurrence of a harpoon-type electron transfer taking place despite the helium environment. To model this process, high-level *ab initio* studies of electronic couplings and the calculation of interaction energies for the relevant electronic states [42] were combined with a concept of solvation-modified reactivity [43]. Specifically, helium density functional theory was employed to account for the extrusion of helium upon the approach of the two reactant species, assuming that the helium droplet behaves as a Bose-Einstein condensate, which can be described with density functional parameters derived from the properties of superfluid bulk helium. Similar to the experiment [25], the simulation showed a high possibility for the formation of a Cs${}_{2}$-C${}_{60}$ complex inside the droplet through a direct harpoon-type electron transfer from Cs${}_{2}$ to C${}_{60}$, but a negligibly low reactivity for atomic Cs. However, the agreement between that model calculation and the experiment also implied that molecular motion inside of a superfluid helium droplet must occur below the critical Landau velocity [44]. These findings motivated us to study the very same reaction mentioned above by means of thermostatted PIMD simulations [40]. In contrast to previous studies, superfluidity is not implicitly assumed, but intrinsically captured by the computational model.

In this work, we provide a mechanistic description of the Cs${}_{2}$ + C${}_{60}$@He${}_{N}$ → Cs${}_{2}^{+}$ + C${}_{60}^{-}$@He${}_{N}$ reaction, with the size of the helium droplet set to N=2090 atoms. As will be discussed below, this is the minimum droplet size necessary for the initial C${}_{60}$-Cs${}_{2}$ distance to be larger than that at the crossing point between the diabatic neutral and ionic potential energy curves at the T-shaped molecular arrangement. The latter geometry is considered as the most probable initial configuration given the conditions of the experimental synthesis. In the NVT ensemble, we consider a temperature of T=2 K. This temperature is lower than the critical value necessary for the onset of helium superfluidity (2.172 K), but is still sufficiently high to render the explicit inclusion of exchange between helium atoms unnecessary [35]. Including exchange, reactive quantum simulations of molecules solvated in superfluid helium were tackled through a remarkable hybrid PIMD/PIMC method [45]. However, the size of the helium droplets was restricted to samples of only 32 and 64 atoms.

This paper is structured as follows. In Section 2, we characterize the potential energy surfaces and provide semiclassical probabilities for the neutral to ionic transition between the Cs${}_{2}$ and C${}_{60}$ dopant species. This is followed by a description of their analytic representations which make the actual calculations feasible. A detailed explanation of the applied PIMD computational method is the subject of Section 3. Next, we present and discuss our results in Section 4. Our main findings are summarized in Section 5.

## 2. Potential Energy Surfaces and Hopping Neutral/Ionic Probabilities

### 2.1. Analytic Potential Energy Surfaces

To make the calculations feasible, we consider the C${}_{60}$ molecule as a structureless point mass and the Cs${}_{2}$ dimer as a rigid rotor with the Cs–Cs distance fixed at its equilibrium value, ${\bar{r}}_{Cs_{2}}\;=\;$6.3 Å. The interactions of the two impurities with helium are assumed to be those of the corresponding neutral species. The total potential can be written as in Equation (1):

(1) $$\begin{array}{c} V^{\left(k\right)}=\sum_{i<j}V_{{\mathrm{He}}_{i}-{\mathrm{He}}_{j}}+\sum_{i}\left[V_{{\mathrm{Cs}}_{2}-{\mathrm{He}}_{i}}+V_{C_{60}-{\mathrm{He}}_{i}}\right]+V_{C_{60}-{\mathrm{Cs}}_{2}}^{\left(k\right)}, \end{array}$$

where k=n stands for the interaction between neutral C${}_{60}$ and Cs${}_{2}$ molecules (referred to as *n*), while k=I refers to the interaction between ionic C${}_{60}^{-}$ and Cs${}_{2}^{+}$ ionic species (referred to as *i*). The He-He interaction in Equation (1) is described via the well-known semiempirical (analytical) form of Aziz and Slaman [46]. It is characterized by a tiny well depth of ∼8 cm${}^{-1}$ at an equilibrium distance of ∼3 Å.

To describe the Cs${}_{2}$-He interaction, we fall back on the *ab initio* points calculated by using the spin restricted single and double excitations coupled cluster method with perturbative triples, Equation (2) [47], via the sum of two angle-dependent Cs-He pair interactions. Each Cs-He interaction is described by a Lennard–Jones function depending on the relative distance *r* and orientation angle ϕ, cosϕ=ρ·r/ρr, with ρ the Cs${}_{2}$ bond-length,

(2) $$\begin{array}{c} V_{\mathrm{Cs}-{\mathrm{He}}_{i}}\left(r_{i},\phi_{i}\right)=d\left(\phi_{i}\right)\left\{{\left[\frac{\bar{r}\left(\phi_{i}\right)}{r_{i}}\right]}^{12}-2{\left[\frac{\bar{r}\left(\phi_{i}\right)}{r_{i}}\right]}^{6}\right\} \end{array}$$

where the ϕ-dependent parameters (energies in cm${}^{-1}$, and distances in Å) in the interval 0≤ϕ≤π/2 are given by Equation (3) [47]

(3) d(ϕi)=0.7+0.3411a(1−cosϕi)4r¯(ϕi)=8.1−0.5431a(1−cosϕi)2,

and a symmetric behavior being assumed in the interval π/2≤ϕ≤π. In this way, the Cs${}_{2}$–He interaction is thus characterized by a well depth of only ∼2 cm${}^{-1}$. The corresponding equilibrium distance (∼6.75 Å) is gradually reduced when going to the collinear arrangement, reaching a value of ∼0.8 cm${}^{-1}$ at a longer distance of ∼11.5 Å (see Figure 1 of Ref. [47]). We notice that the heliophobic character of the Cs${}_{2}$ dimer is a direct consequence of the difference between the Cs${}_{2}$-He and the He-He interactions mentioned above. Each C${}_{60}$-He pair interaction was fitted [43] to a potential form as a function of the relative distance (*s*) using the *ab initio* values calculated via symmetry-adapted perturbation theory, Equation (4) [48],

(4) $$\begin{array}{c} V_{C_{60}-{\mathrm{He}}_{i}}\left(s_{i}\right)=\sum_{j=3}^{6}F_{2j}/s_{i}^{2j}. \end{array}$$

The C${}_{60}$-He pair interaction is characterized by a well depth of ∼56 cm${}^{-1}$ at a distance of ∼6.8 Å (see Figure 4 of Ref. [43]). It is thus much stronger than the He-He interaction (ca. 8 cm${}^{-1}$), explaining the heliophilic nature of the fullerene molecule. The *F* parameters (see Equation (4)) were obtained through a nonlinear least squares fitting and are listed in Table 1.

Finally, the Cs${}_{2}$-C${}_{60}$ and Cs${}_{2}^{+}$-C${}_{60}^{-}$ interactions need to be described as functions of the relative distance *R* and orientation θ, cosθ=ρ·R/ρR. Here, we employ *ab initio*-generated potential energy curves in the diabatic representation for both collinear (C) and T-shaped (T) arrangements (see Ref. [42]). The potential energy curves reported in Ref. [42] were further improved through a counterpoise correction, which was applied as described in the Supporting Information of Ref. [42].

Highly accurate studies of alcaline metal-contained systems showed the importance on including relativistic corrections (see, e.g., [49]). To make the calculations feasible and mimic relativistic effects, we used the ECP46MDF effective core potential of the Stuttgart/Köln group [50]. Second-order Douglas-Kroll-Hess Coupled-Cluster calculations [51] on the Ag${}_{2}$-benzene interaction energy (i.e., including relativistic corrections) showed that the error introduced by the relativistic pseudopotential approximation is below 0.5% [52]. We can expect a similar order of magnitude for the case of the Cs${}_{2}$-C${}_{60}$ system.

The interactions at any given orientation can be inferred from those at C and T configurations using the formula written in Equation (5):

(5) $$\begin{array}{ccc} V_{C_{60}-{\mathrm{Cs}}_{2}}^{\left(k\right)}\left(R,\theta \right) & = & \{\left[V_{C}^{\left(k\right)}\left(R\right)+2V_{T}^{\left(k\right)}\left(R\right)\right]+\\ & & \left[V_{C}^{\left(k\right)}\left(R\right)-V_{T}^{\left(k\right)}\left(R\right)\right]\left(3cos^{2}\theta -1\right)\}/3. \end{array}$$

Introducing the placeholder *o* to denote the collinear (o=C) or the T-shaped (o=T) orientations, the following analytical forms written in Equation (6) are used,

(6) $$\begin{array}{c} V_{o}^{\left(k\right)}\left(R\right)=\left\{\begin{array}{cc} f_{o}^{\left(k\right)}\left(R^{\prime }\right)\sum_{j=1}^{7}\frac{A_{2j}^{o}}{{\left(R^{\prime }\right)}^{2j}}+{\tilde{f}}_{o}^{\left(k\right)}\left(R^{\prime }\right)\frac{C_{6}}{R^{\prime 6}}, & k=n\\ f_{o}^{\left(k\right)}\left(R\right)\sum_{j}\frac{B_{j}^{o}}{{\left(R+q_{j}^{o}\right)}^{j}}+{\tilde{f}}_{o}^{\left(k\right)}\left(R\right)\left(D_{0}-\frac{D_{1}}{R}\right), & k=I, \end{array}\right. \end{array}$$

where $f_{o}^{\left(k\right)}\left(R\right)=\left\{1-\tanh\left[\left(R-R_{o}^{\left(k\right)}\right)/2\right]\right\}/2$ and ${\tilde{f}}_{o}^{\left(k\right)}=1-f_{o}^{\left(k\right)}$ functions softly connect the regions with $R\leq R_{o}^{\left(k\right)}$ and $R\geq R_{o}^{\left(k\right)}$ at the matching points $R_{o}^{\left(k\right)}$. In the first row of Equation (6), $R^{\prime }=R$ for all angular orientations with the exception of the collinear approach. In the latter case, a shift $R^{\prime }=R-{\bar{r}}_{Cs_{2}}/2$ occurs since the *ab initio* interaction is described as a function of the distance from the C${}_{60}$ molecule to the nearest Cs atom. The sum appearing in the second row of Equation (6) runs over the values j=0,1,6, and 12. The necessary parameters were obtained through nonlinear least squares fitting and are also listed in Table 1.

**Figure 1:**
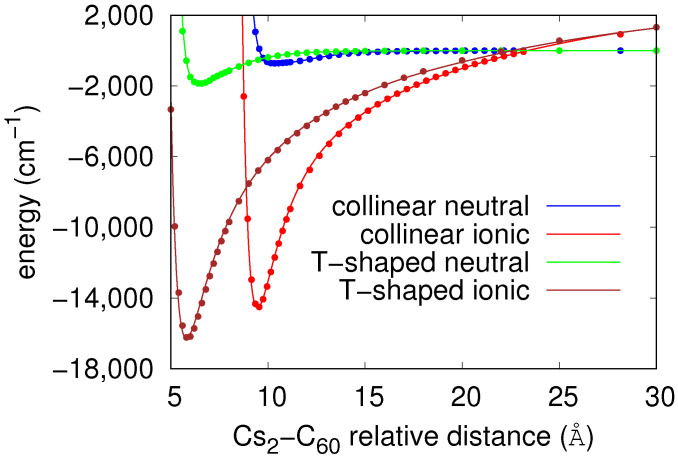
Diabatic potential energy curves characterizing the neutral Cs${}_{2}$-C${}_{60}$ and ionic Cs${}_{2}^{+}$-C${}_{60}^{-}$ pair interactions as function of their relative distance *R* at collinear and T-shaped orientations. Points: *ab initio* calculations; solid lines: analytic fits, see text.

Figure 1 shows the diabatic potential energy curves of neutral Cs${}_{2}$-C${}_{60}$ and ionic Cs${}_{2}^{+}$-C${}_{60}^{-}$ systems at the extreme *C* and *T* orientations. Notice the accuracy of the analytical fits when compared to that of the *ab initio* values. As expected, the well depth of the potential describing the ionic Cs${}_{2}^{+}$-C${}_{60}^{-}$ interaction is deeper than the potential characterizing the dispersion-dominated interaction between neutral Cs${}_{2}$ and C${}_{60}$ species, with the *T* orientation being more stable than the *C* orientation. At the *T*-shaped configuration, the ionic curve shows a well depth of 16,265 cm${}^{-1}$ at a relative distance of 5.76 Å, while, for the neutral curve, these values are 1878 cm${}^{-1}$ and 6.42 Å, respectively. The two potential energy curves cross at a distance of 22.57 Å. For the collinear configuration, the well depth and equilibrium distance is 14,570 cm${}^{-1}$ at 9.46 Å (ionic) and 718 cm${}^{-1}$ at 10.42 Å (neutral), with the crossing located at 23.35 Å.

**Figure 2:**
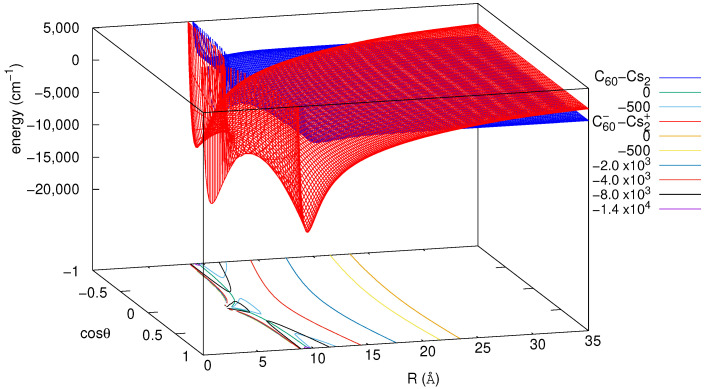
Analytic potential energy surfaces characterizing neutral Cs${}_{2}$-C${}_{60}$ and ionic Cs${}_{2}^{+}$-C${}_{60}^{-}$ pair interactions as a function of their relative distance *R* and orientation θ.

Figure 2 shows both the ionic and the neutral potential energy surfaces as they are obtained by application of Equation (5). As can be seen, the PESs cross at distances close to ∼23 Å, depending on θ. Interestingly, it can be observed in Figure 2 that the valley is much narrower for the T-shaped configuration than it is for a collinear orientation. As a result, a simple 2D rotationless (i.e., with total angular momentum *J* = 0) bound-state calculation [53] for the isolated ionic Cs${}_{2}^{+}$-C${}_{60}^{-}$ system already shows that the 2D ground-state energy level (∼14,470 cm${}^{-1}$) as well as the first vibrationally excited bound states correspond to a mostly collinear configuration. Contrarily, the binding energy of the corresponding 1D potentials is larger for the T-shaped (16,250 cm${}^{-1}$) than for that of the collinear configuration (14,543 cm${}^{-1}$).

### 2.2. Hopping Neutral/Ionic Probabilities

Similar to Ref. [42], the electron hopping probabilities between the neutral and ionic state is estimated by resorting to the semiclassical Landau–Zener model using the expression written in Equation (7),

(7) $$\begin{array}{c} P\left(v\right)\approx 1-\exp[\left(-2\pi H_{12}^{2}\right)/\left(\hbar \Delta F_{12}v\right)], \end{array}$$

with *v* denoting the Cs${}_{2}$-C${}_{60}$ relative velocity and $H_{12}$ the off-diagonal matrix element of the electronic Hamiltonian. The latter is computed by explicitly using the electronic wavefunctions in a diabatic representation, and $\Delta F_{12}$ is the absolute value of the difference between the slopes of the two diabatic potential energy curves. All these values are evaluated at the intersection between the two potential energy curves. The approach adopted here assumes that the diabatic potentials, as well as the corresponding couplings, are those of the gas phase, i.e. unperturbed by the helium environment. The technical procedure to calculate diabatic neutral and ionic potential energy surfaces as along with the corresponding electronic couplings was extensively described in Section S1 of the Supporting Information of Ref. [42]. Specifically, a similar strategy implemented in Refs. [54,55] to the charge transfer from reduced TiO${}_{2}$ surfaces to molecular oxygen was followed. It employs orbitals separately optimized for neutral and ionic electronic states to render the calculations feasible in large systems. In the present case, the interaction energies were obtained at second-order Möller–Plesset perturbation theory (MP2) and the diabatization procedure consisted in rotating the two states at a given geometry in such a way that their overlap with the diabatic states at the next larger distance is maximized.

**Figure 3:**
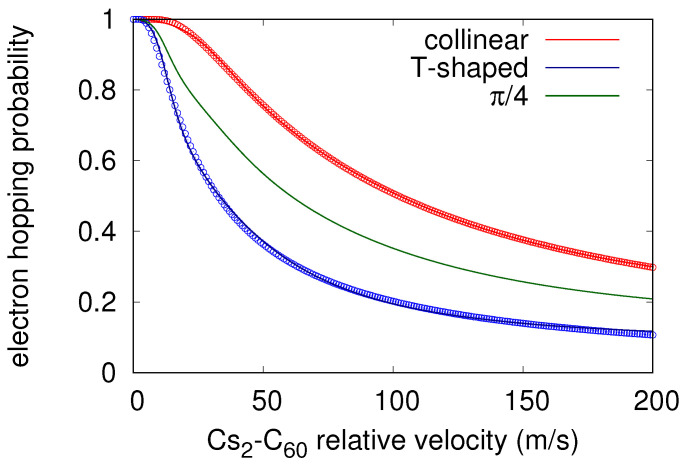
Computed (points) and fitted (lines) of probability for electron hopping obtained using the Landau–Zener model at collinear and T-shaped orientations as function of relative velocity [42]. Simple analytic extension at whatever be the orientation is also shown for an intermediate orientation θ=π/4.

Figure 3 presents the probabilities of electron hopping (circles) at the crossing points for both collinear and T-shaped configurations, as estimated according to Equation (7). The figure also contains analytical fits for the two extreme configurations (solid lines), based on the expression Equation (8)

(8) $$\begin{array}{c} P\left(v\right)=a/\left(b+v^{2}\right)+c/\left(d+v^{4}\right)+\left(1-a/b-c/d\right). \end{array}$$

The values of the orientation-dependent parameters are listed in Table 2. Using the values of the probabilities for collinear and T-shaped configurations, the electron hopping probability can be expressed as a function of the orientation θ, Equation (9):

(9) $$\begin{array}{c} P\left(v,\theta \right)=\{\left[P_{C}\left(v\right)+P_{T}\left(v\right)\right]+\left[P_{C}\left(v\right)-P_{T}\left(v\right)\right]\left(3cos^{2}\theta -1\right)\}/3. \end{array}$$

As an illustrative example, the interpolated hopping probability P(v,π/4) is shown in Figure 3. It almost reaches a value of one at the collinear orientation for relative velocities ≤ 25 m/s, and decreases to 60% when going towards the T-shaped arrangement. However, for relative velocities below 12.5 m/s the probability reaches values ≥80% independent of the orientation.

## 3. Computational Method and Details

### 3.1. Initial Arrangement

**Figure 4:**
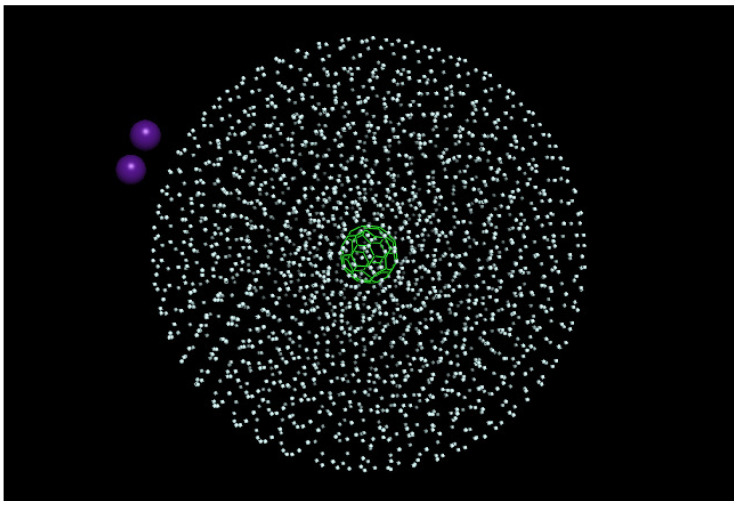
Initial structure of Cs${}_{2}$-C${}_{60}$@He${}_{2090}$ complex obtained by a combined evolutionary algorithm and geometric extension, see text.

To start with the simulations, we define an initial He matrix configuration by applying an evolutionary algorithm [33] to C${}_{60}$@He${}_{N}$ complexes. The algorithm works properly up to N=300, with the spherically-shaped complex reaching a radius of ∼12 Å. However, beyond N=300, this approach becomes increasingly inefficient. Hence, since the C${}_{60}$-He interaction is negligible for larger distances, and since the He-He equilibrium distance is ∼π Å, He droplets with N≥300 are modeled through a simple geometric extension, where successive spheres of radius 12+πn Å, n=1,⋯,4, are filled with He atoms. To this end, semicircumferences of radius 12+πn Å are first filled with an integer number of He atoms. For a given semicircumference, each He atom is located at the equilibrium He−He distance (∼π Å) from each other. Next, we apply a full rotation around the corresponding diameter. In this way, each atom describes a circumference of known radius (i.e., the corresponding C${}_{60}$-He distance). Finally, each circumference is filled with He atoms by following the same criteria (i.e., locating each He atom at a distance ∼π Å from each other). As a result, for n=4, we obtain a C${}_{60}$@He${}_{2090}$ complex with a radius of ∼24 Å. For this choice of droplet size, by placing the Cs${}_{2}$ dimer near its surface, the distance between the Cs${}_{2}$ and C${}_{60}$ impurities becomes slightly larger than the crossing distance between the corresponding diabatic neutral and ionic potential energy curves for the T-shaped orientation. Once again, the T-shaped configuration is assumed to be the more probable initial arrangement due to the characteristics of the experimental set-up. The actual structure of the total system is illustrated in Figure 4 for the Cs${}_{2}$-C${}_{60}$@He${}_{2090}$ complex. This structure is adopted as the initial one for all the simulations performed.

### 3.2. PIMD Simulation

For a given potential energy model, PIMD simulations in the NVT ensemble provide an approximate description of quantum fluctuation effects of the nuclei in static equilibrium properties. The path integral calculation can be thermostatted through several schemes [40]. To describe approximate quantum dynamics in the NVE ensemble, the Ring Polymer Molecular Dynamics [56] (RPMD) approach is defined as evolving under the same choice of Hamiltonian as for the PIMD implementation but without turning on the thermostats. However, due to the ergodicity problems associated with the path integral Hamiltonian, it is necessary to launch trajectories with many different choices of the initial momenta. In other words, it is required to do a NVT thermostatted PIMD simulation and, next, to launch many NVE RPMD trajectories from the configurations generated in the PIMD run. In this work, we mainly addressed the first issue, i.e., the thermostatted NVT simulations, using the i-PI open code of Ceriotti et al. [57], using the analytical potential model described above. To assess the possible incidence of the thermostat by masking the time evolution of the system, real time NVE simulations were additionally carried out; see SI appendix.

Based on the white noise Langevin thermostat, we use the global version of the path integral Langevin equation (PILE-G) stochastic thermostatting scheme [40] with a unique input parameter $\tau_{0}$, the friction coefficient determining thermostat efficiency. For a temperature of 2 K, we considered a value of $\tau_{0}=5$ fs throughout all simulations. Using a large cubic cell with a box width of 125 Å, it is not necessary to incorporate barostats since the pressure always remains close to zero. The chosen time interval (Δt=0.2 fs) is approximately a tenth of the smallest period in the physical system (∼2 fs). It corresponds to the maximum kinetic energy of the Cs${}_{2}^{+}$-C${}_{60}^{-}$ interaction, ∼16,265 cm${}^{-1}$ for the T-shaped orientation, see the potential energy minimum in Figure 1. We controlled the quality of the simulation through the so-called effective energy [58] in addition to the temperature. After a period of stabilization (typically ∼1–2 ps), the latter oscillates around 2 K within 0.1 K while the former is kept unmodified within a variation of ∼0.1%. Due to the huge number of particles, the considered numbers of beads *M* in the extended system (ring polymer) were modest (M=1, 5, and 10) but large enough to enable the description of quantum effects. As explained in SubSection 3.1, the initial configuration of the complex was obtained by combining an evolutionary algorithm [33] and a geometric extension. This measure leads to a structure in which the Cs${}_{2}$ impurity is close to the surface of the droplet in a near T-shaped configuration. Initial velocities were generated both from a Maxwell–Boltzmann distribution at the given temperature and multiplying the previous momenta corresponding to the different beads of cesium atoms by factors of 1.5, 2.5, 3.5, and 4.5, which increases the initial relative Cs${}_{2}$-C${}_{60}$ velocity, with the C${}_{60}$ molecule remaining immobile. All the magnitudes were estimated within the centroid approximation.

## 4. Results

We start the simulation with the system on the neutral potential energy surface, with all atoms distributed according to the structure shown in Figure 4. Using the i-PI code, the success of the redox reaction is estimated by comparing the Landau–Zener probability with a random number along a large batch of runs. However, we realized that this procedure can become very inefficient and time consuming as a huge number of comparisons should be done. Instead, as explained below, we resort to *a posteriori* procedure. So, just a few runs are carried out with different, feasible levels of quantization, M=1 (classical), 5, and 10, considering different values of the initial relative Cs${}_{2}$-C${}_{60}$ velocity. These initial values were $v_{0}=25.4$ m/s, stemming from a Maxwell distribution at T=2 K for all the *M* values, and, as mentioned above, those obtained by multiplying the corresponding previous momenta of all the beads describing the Cs atoms by factors of 1.5, 2.5, 3.5, and 4.5. In the first four cases no reaction was attained and so we only show the results coming from the thermal initial velocity. For the latter case, factor 4.5, this procedure leads to a second set of initial velocities $v_{0}$ of 129.1 m/s (M=1), 169.4 m/s (M=5), and 239.8 m/s (M=10), and a reaction is observed. As shown below, considering the small value of the initial velocity (25.4 m/s), and whatever the level of quantization be, the Cs${}_{2}$ is unable to overcome the hindrance due to the presence of He atoms and never reaches the intersection with the ionic state, i.e. the reaction does not take place. On the contrary, when the initial values of the velocities are increased, the intersection distance between neutral and ionic states is reached (tolerance of 0.1 Å). Then, the jump of one electron from the Cs${}_{2}$ dimer to the Cs${}_{60}$ molecule occurs and the system continues to evolve on the ionic surface. We estimate the probability of the redox process *a posteriori* by analyzing the relative Cs${}_{2}$-C${}_{60}$ velocity as far as the reactant species reach the intersection distance.

**Figure 5:**
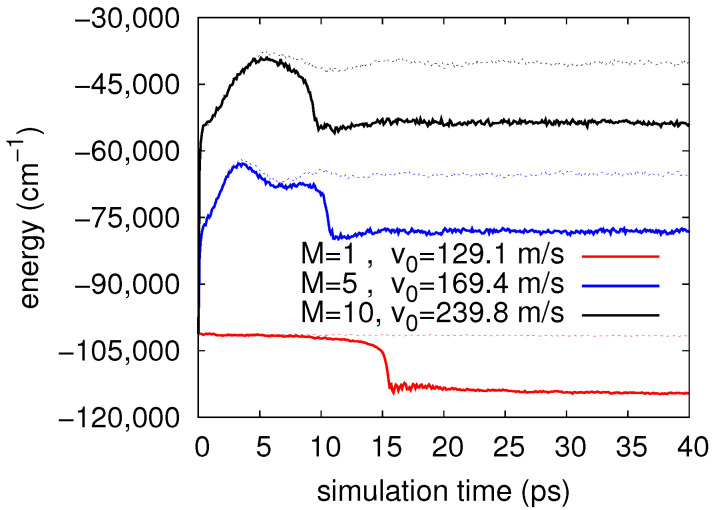
Time evolution of the total energy of the Cs${}_{2}$-C${}_{60}$@He${}_{2090}$ system at T=2 K, obtained in simulations performed at the indicated number of beads *M*. Solid lines correspond to initial relative velocities of the Cs${}_{2}$-C${}_{60}$ interacting pair ($v_{0}$) indicated in the figure, while dashed lines, maintaining the relation color/*M*, correspond to a common initial velocity $v_{0}=25.4$ m/s.

Figure 5 shows the energy evolution as a function of time (up to 40 ps) for the different scenarios considered here: starting with the same initial configuration, i.e., a doped C${}_{60}$@He${}_{2090}$ droplet with the fullerene in its center plus the Cs${}_{2}$ dimer in an approximate T-shaped orientation with respect to the C${}_{60}$ molecule and at a distance R=23 Å on the neutral surface, we carried out PIMD simulations at T=2 K accounting for M=1, 5, and 10 beads. Considering an initial common relative velocity $v_{0}=25.4$ m/s, stemming from a Maxwell distribution at T=2 K, the energy evolution are indicated with dashed lines. For M=1 (classical simulation) the energy remains almost constant at a value of ∼−100,000 cm${}^{-1}$ reflecting that the system is kept very close to its initial configuration ever and ever. For M=5 and 10, the corresponding energies increase within in the first 5 ps and then remain almost constant, showing only minimal oscillations. As *M* increases, the final energies approach gradually the zero point energy of the C${}_{60}$@He${}_{2090}$ system plus the small contribution from Cs${}_{2}$ attached to its surface; however, a full convergence of this energy is not the goal of the present study. When the precedent initial momenta corresponding to the beads associated to cesium atoms are multiplied by a constant factor of 4.5, as was already mentioned in Section 3, the initial relative velocities between the two reactants estimated in the centroid approach increase to 129.1 m/s, 169.4 m/s, and 239.8 m/s for M=1, 5, and 10, respectively. The results are plotted as solid lines in Figure 5. For M=1, a sudden decrease in energy of ∼14,000 cm${}^{-1}$ is observed at ∼15 ps of simulation. After some oscillating increases, similar jumps are produced before (∼10 ps) for M=5 and 10, but the final distance between the corresponding dashed and solid lines remains of the same magnitude of ∼14,000 cm${}^{-1}$ (which corresponds to the linear well in Figure 1) indicating that there was a transition from the neutral T-shaped state to the ionic collinear one, i.e., the charge transfer took place involving a reorientation between the reactants. This will be corroborated below when analyzing the evolution of distances and orientation of dopants over time.

**Figure 6:**
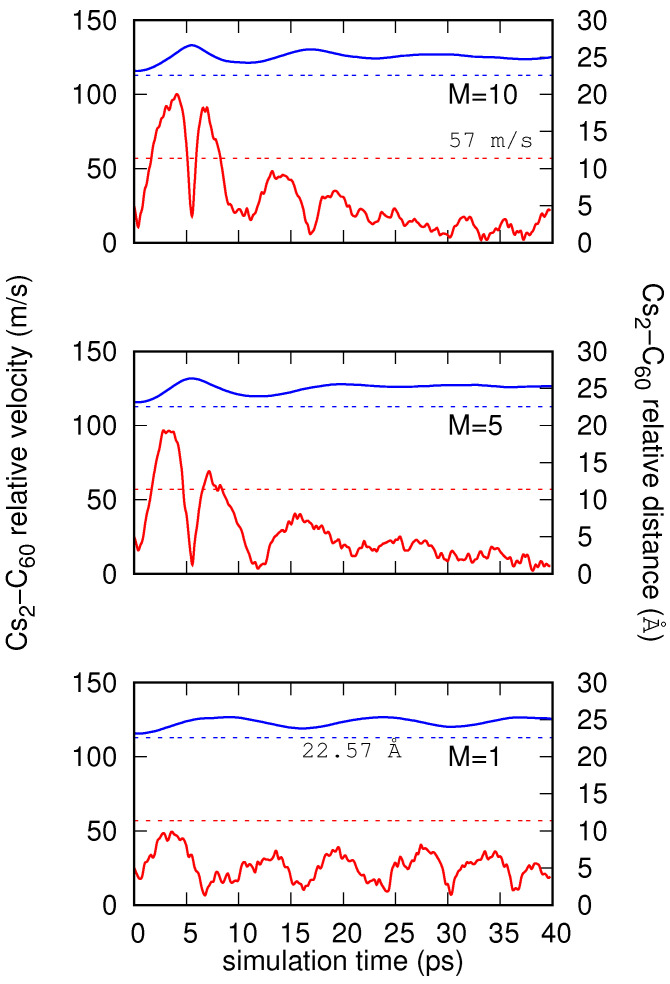
Evolution of relative Cs${}_{2}$-C${}_{60}$ distance (blue solid lines, right vertical axis) and velocity (red solid lines, left vertical axis) as a function of time. It was obtained through PIMD simulations for number *M* of beads considered, with common initial relative velocity being $v_{0}=25.4$ m/s. Constant dashed lines indicate the critical Landau velocity (shown in red) as well as the crossing distance between neutral and ionic potential energy curves at initial T-shaped orientation (shown in blue).

The three panels of Figure 6 shows the evolution of relative Cs${}_{2}$-C${}_{60}$ distances and velocities as a function of time for the common initial velocity $v_{0}=25.4$ m/s and M=1 (lower panel), 5 (medium panel), and 10 (top panel). They were estimated in the centroid approximation. In agreement with the evolution of energy shown in the previous figure, and in all cases, the distances remain above the crossing point of the neutral/ionic curves near the T-shaped arrangement (22.57 Å). In other words, an intersection of potential curves is never reached, and consequently the charge transfer does not take place. The droplet keeps the fullerene fully immersed, while the cesium dimer remains on its surface with negligible interaction between the reactants. The relative velocities for M=1 remain always below the Landau velocity. For M=5 and 10, this is true from ∼10 ps of simulation. The sudden jumps of velocities for M=5 and 10 in the neighborhood of 5 ps correspond to a rebounce of the cesium on the surface of the droplet.

**Figure 7:**
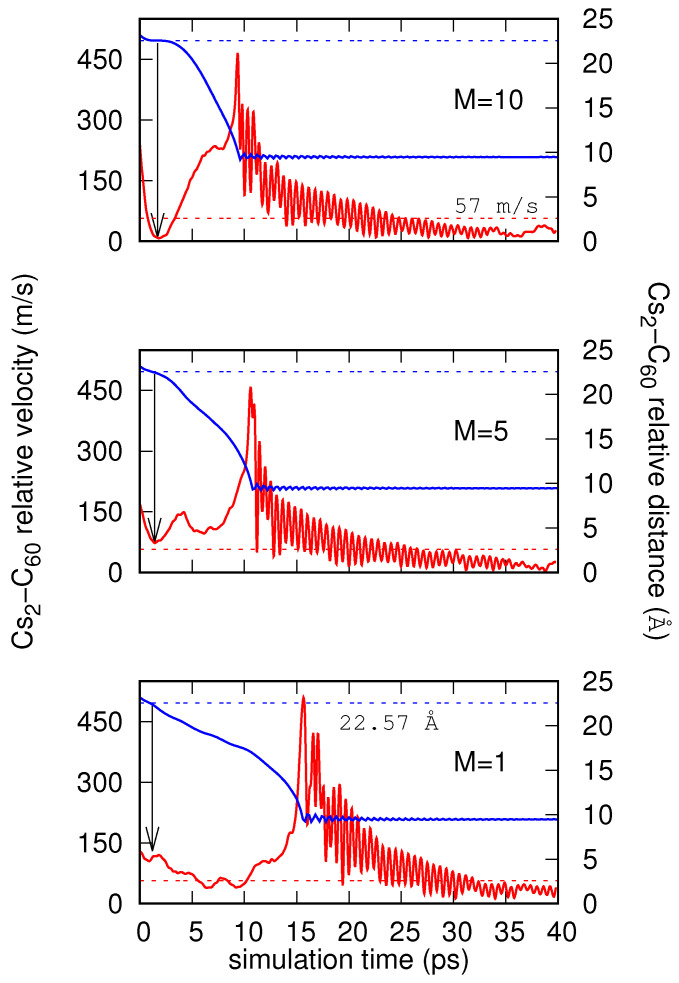
Evolution of relative Cs${}_{2}$-C${}_{60}$ distance (blue solid lines) and velocity (red solid lines), plotted as a function of time, and obtained through simulations for *M* beads (M=1, 5, and 10) but with different initial relative velocities of $v_{0}=129$ m/s (M=1), 169.4 m/s (M=5), and 239.8 m/s (M=10). Landau critical velocity and crossing distance between neutral and ionic potential energy curves at T-shaped initial orientation are also shown with dashed red and blue lines, respectively. Arrows indicate relative velocity of reactants when they get crossing distance between neutral and ionic potential energy curves.

Similar to Figure 6, the three panels of Figure 7 show the time evolution of the relative Cs${}_{2}$-C${}_{60}$ distances and the relative velocities, considering the initial velocities $v_{0}=129.1$ m/s (M=1, lower panel), 169.4 m/s (M=5, medium panel), and 239.8 m/s (M=10, top panel). In agreement with the evolution of energies shown in Figure 5 with solid lines, from ∼2 ps, the values of the relative Cs${}_{2}$-C${}_{60}$ distance show a quick decrease (up to ∼15 ps for M=1 and ∼10 ps for M=5, 10) and then stabilize with small oscillations around a value of ∼9.5 Å. This latter value of the relative distance (9.5 Å) corresponds to the equilibrium distance of the ionic Cs${}_{2}^{+}$–C${}_{60}^{-}$ system at the collinear configuration. Note that when reaching the intersection value of 22.57 Å, the slope of the distance as a function of time decreases as *M* increases. In particular, for M=10, there is a clear plateau at the intersection value that enhances the probability of jump from the neutral to the ionic state of the Cs${}_{2}$ and C${}_{60}$ impurities. As for the relative velocities (see the arrows on the figure), the M=1 case presents a value close to 150 m/s at the crossing, while this value is ∼75 m/s for M=5, and ≤10 m/s (well below the Landau velocity) for M=10. Such velocities reach minima which reflect the braking produced by the presence of the He atoms. By considering these velocities, and according to the Landau–Zener probabilities shown in Figure 3, we can assign a probability of reaction of 10% for M=1, increasing as *M* increases to 20% for M=5, and above 95% for M=10. In other words, the reaction is favored when the quantum nature of the He atoms is accounted for with increasing accuracy. For all the *M* values, after the jump from the neutral to the ionic state occurs and the relative Cs${}_{2}$-C${}_{60}$ distance decreases, the relative velocity increases up to a maximum when the Cs${}_{2}$-C${}_{60}$ distance gets the equilibrium value for the collinear arrangement. Then, although with marked oscillations, the relative velocity quickly decreases, reaching values clearly below the critical Landau velocity. However, it was pointed out that the flow of a uniform Bose gas at speeds greater than the critical Landau velocity $v_{c}$ does not necessarily destroy superfluidity [59], but rather needs only to lead to a decrease of the superfluid mass density $\rho_{s}$.

**Figure 8:**
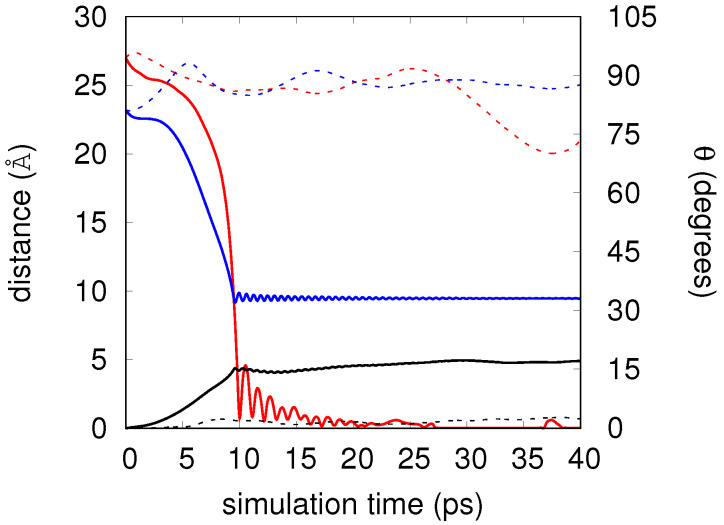
For M=10, evolution of relative Cs${}_{2}$-C${}_{60}$ distance, *R* and orientation, θ, as function of time. Dashed lines correspond to an initial velocity $v_{0}=25.4$ m/s, while solid lines show simulation starting with $v_{0}=239.8$ m/s. Blue lines: relative distance between two reactants; red lines: relative orientation; black lines: distance from C${}_{60}$ to its original position at time 0.

For M=10 and initial velocities $v_{0}=25.4$ (dashed lines) and 239.8 m/s (solid lines), Figure 8 shows the evolution of the relative Cs${}_{2}$-C${}_{60}$ distance as a function of time (blue lines as already shown in the upper panels of Figure 6 and Figure 7) and also the distance from C${}_{60}$ to its original position at time 0 (black lines), together with the orientation θ of Cs${}_{2}$ with respect C${}_{60}$ (red lines, right vertical axis) for the two velocities. For the lower velocity, the orientation remains oscillating around 90 degrees showing that the cesium dimer, always remaining on the surface of the droplet, performs a nodding in addition to the main rotation on the plane parallel to the surface of the droplet. For the higher velocity, at which the reaction takes place with a probability of almost unity, and according to the evolution of the relative distance, the orientation goes from perpendicular to collinear as the relative distance drops off within ∼10 ps, and then presents decreasing oscillations to finish in this last configuration. Black lines reflect the mobility of fullerene along the simulation. For the lower initial velocity, the heliophilic dopant remains always close to its original position. The same holds for the upper velocity until the charge transfer begins (∼2 ps) and then increases up to ∼5 Å at ∼10 ps, remaining constant from here on. Hence, and as could be expected because of the characteristics of the interaction, the simulations show a rather small mobility of C${}_{60}$ inside the droplet.

**Figure 9:**
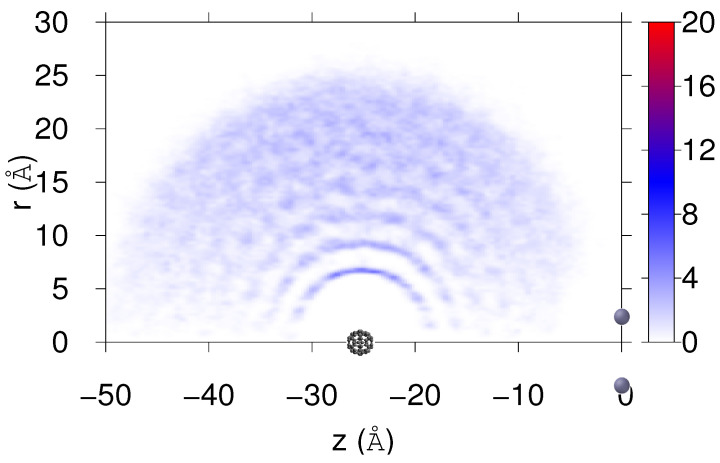
For M=10 and $v_{0}=25.4$ m/s, and using cylindric coordinates with *z*-axis in direction of line joining Cs${}_{2}$ and C${}_{60}$ mass centers, final He density distribution ($\times 10^{-3}$ Å${}^{-2}$). Positions of Cs${}_{2}$ and C${}_{60}$ impurities are also indicated.

Using cylindric coordinates with the *z*-axis in the direction of the line joining C${}_{60}$ and Cs${}_{2}$ centers of mass, with the latter defining the origin, Figure 9 shows the density probability of He atoms. This probability was estimated by accumulation (and final renormalization) from the simulation performed for M=10 and $v_{0}=25.4$ m/s within the interval [10–40] ps. The positions of the Cs${}_{2}$ and C${}_{60}$ impurities are also indicated in the figure. The quasispherical helium distribution with a radius of about 25 Å is barely distorted by the adsorbed Cs${}_{2}$ impurity. The fullerene molecule is located in the center, while the cesium dimer is unable to penetrate and remains outside in a T-shaped configuration, producing a small dimple on the surface of the droplet.

**Figure 10:**
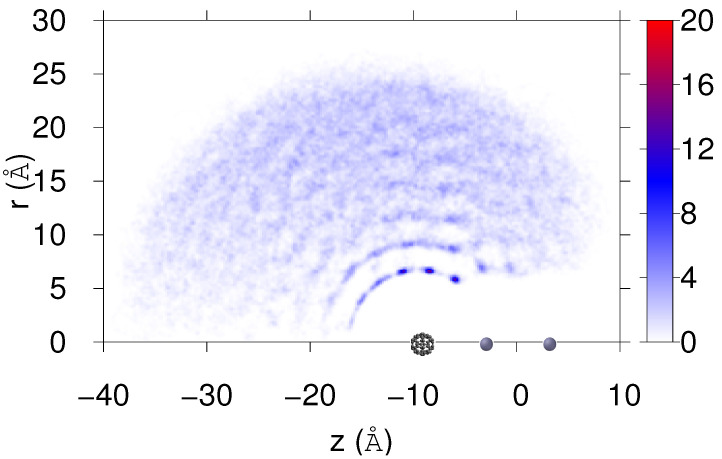
Final He density distribution ($\times 10^{-3}$ Å${}^{-2}$) in cylindric coordinates for M=10 when considering an initial relative velocity $v_{0}=239.8$ m/s. Positions of Cs${}_{2}$ and C${}_{60}$ impurities are also indicated.

**Figure 11:**
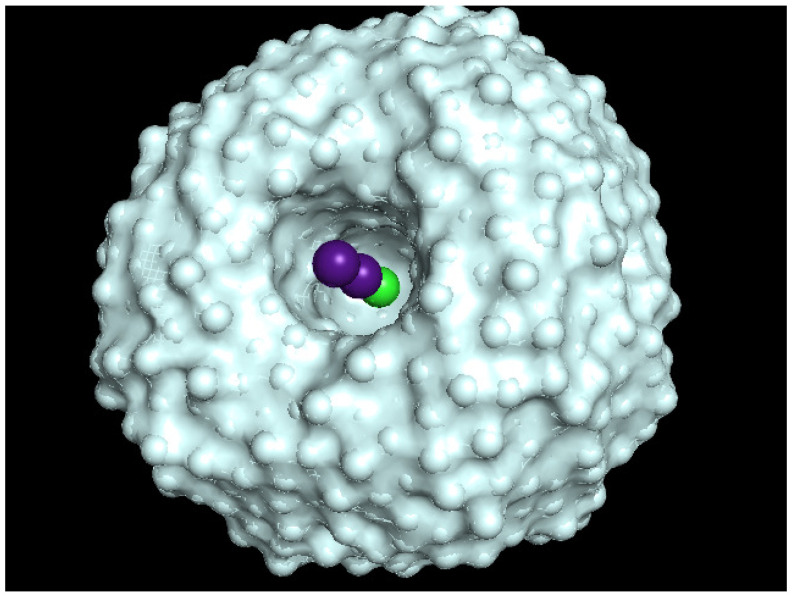
Structure of Cs${}_{2}$-C${}_{60}$@He${}_{2090}$ system at 40 ps from a simulation with M=10 and $v_{0}=239.8$ m/s.

In Figure 10, the final helium density distribution is shown as obtained from the simulation performed for M=10 but with initial velocity $v_{0}=239.8$ m/s. Notice that the impurities are now in a collinear configuration, and that the initial quasispherical structure becomes clearly distorted: the cesium dimer opens a tunnel in the droplet to meet the fullerene molecule, and a considerable amount of He migrates to the opposite side. A snapshot of this situation is shown in Figure 11 for the last structure obtained at the end of the simulation, i.e., 40 ps.

**Figure 12:**
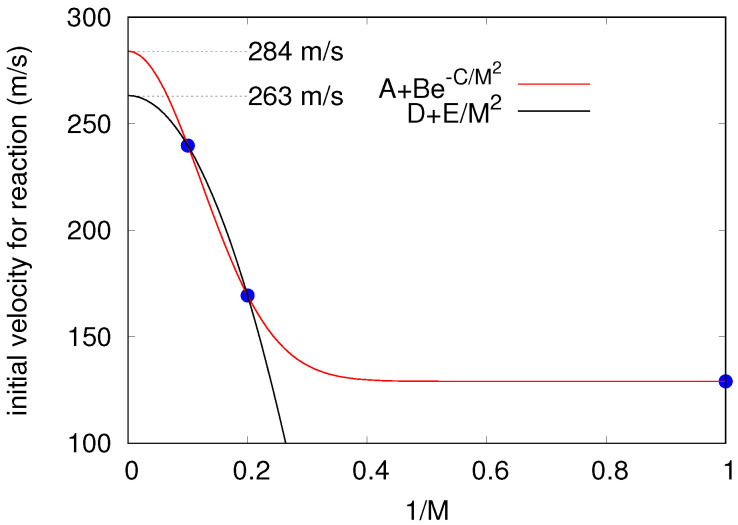
Extrapolation of initial velocity allowing for electron hopping to M→∞.

Summarizing, multiplying the momenta of the beads associated to the Cs atoms by a constant factor of 4.5 the initial velocity grows to values from 129.1 m/s (M=1) to 239.8 m/s (M=10). At these velocities the reaction takes place, with the probability becoming larger as the quantization level increases from 10% (M=1) to 95% (M=10). Although for M=1 the velocity of 129.1 m/s is close to the threshold leading to reaction, we do not state that the same holds true for the corresponding values for M=5 and 10. Probably slightly lower values (higher than 3.5) also allow for the redox reaction. Anyway, assuming that the values employed are reactivity thresholds, we are aware that they increase with *M* in such a way that it is interesting to estimate the value reached when M→∞. To this end, Figure 12 shows two extrapolations. The first extrapolation was achieved by fitting the values obtained for M=5 and 10 to the quadratic form also employed to extrapolate energies [60]. The second extrapolation consists in fitting the values calculated for M=1, 5, and 10 to a Gaussian function in terms of 1/$M^{2}$. The extrapolated values define an interval from 263 to 284 m/s, and these subsonic velocities are acceptable as long as the cesium dimer is already formed when it becomes picked up by the traveling droplet. Otherwise, with the cautions inherent to the treatment applied here, if the cesium dimer is formed by skating (cold collisions) of cesium atoms on the surface of the aggregate [18,23], one would have to assume a high mobility of the fullerene inside the droplet for approaching the surface, as stated in the experimental work [25]. This scenario is not apparent at all in our atomistic study. However, the energy released upon Cs${}_{2}$ formation could suffice to excite the droplet and produce an out-of-equilibrium situation in which either the Cs${}_{2}$ dimer would have enough kinetic energy to overcome the energetic barrier from the extrusion of helium atoms, rendering its approach to the fullerene molecule feasible. Alternatively, the fullerene molecule could be excited from its center-of-the-droplet position towards the droplet surface and then react with the Cs${}_{2}$ dimer. In other words, alternative reaction mechanisms are thinkable if the constraint of a fully equilibrated system is removed. The first possibility, i.e., the Cs${}_{2}$ formation on the surface of the droplet, appears to be more realistic. On one hand, no dependence on the number of C${}_{60}$ molecules was found in the experiment [25]. On the other hand, a larger number of C${}_{60}$ molecules would enhance the probabilities of reaching closer positions to the surface of the droplet. Moreover, the HND’s surface tension is expected to decrease as the HND’s size increases, with the the droplet produced in the experiment being a factor of 50 larger than that of those considered in our simulation. In short, a reduced energetic barrier and threshold velocity is expected for Cs${}_{2}$ submersion into larger He droplets. For the sake of completeness, the values of the extrusion barrier for the Cs${}_{2}$ dimer (in a T-shaped configuration) was also calculated via dispersion-corrected DFT calculations of the C${}_{60}$@He${}_{300}$-Cs${}_{2}$ reaction pathway (i.e., beyond the pairwise approximation for the interaction potential), being 2000 cm${}^{-1}$ at the surface of the He${}_{300}$ droplet (at 12 Å from the C${}_{60}$ center-of-mass).

### Real-Time Simulations in the NVE Ensemble

To deliver results in real-time, illustrative PIMD calculations in the NVE microcanonical ensemble were carried out as well (see the Supplementary Material). Similar conclusions were reached than in the NVT simulations. In particular, the NVE simulations confirm that higher velocities than the thermal one are necessary to overcome the energetic barrier from the extrusion of helium atoms. Also, the mobility of the C${}_{60}$ species remains being quite small: it departs no more than 5 Å from its initial position. The evaporation of helium atoms due to the reaction heat is also apparent when the level of quantization increases (for M=10, see also Video S1). Confirming the *ab initio* predictions presented in Ref. [42], it also nicely shows how the Cs${}_{2}$ dimer rotates before the charge transfer process occurs, with one He atom evaporating when the Cs${}_{2}$ dimer becomes bonded to the fullerene molecule. In fact, once the charge transfer happens, a large amount of energy is distributed over the kinetic energy of the helium atoms, which subsequently leads to atomic helium evaporation (see, e.g., Ref. [61]).

## 5. Conclusions

To study the charge transfer reaction Cs${}_{2}$-C${}_{60}$@He${}_{N}\to$ Cs${}_{2}^{+}$-C${}_{60}^{-}$@He${}_{N}$ from a mechanistic point of view, thermostatted PIMD simulations at T=2 K in the NVT ensemble were carried out using the i-PI open code of Ceriotti et al. [57]

The cesium dimer was considered as a rigid rotor and the fullerene as a mass point. The relevant neutral and ionic potential energy surfaces were described as simple addition of subunits including anisotropy effects. The He-He interaction was described through the semiempirical form of Aziz and Slaman [46], and the other pair interactions were analytically fitted to previous high-level *ab initio* calculations [42,43,47,48]. Two extreme orientations, collinear and T-shaped, were initially considered for the description of Cs${}_{2}$-C${}_{60}$ and Cs${}_{2}^{+}$-C${}_{60}^{-}$ interactions, and then interpolated for whatever the configuration be. A semiclassical Landau–Zener hopping model was applied to estimate the reaction probabilities [42].

To include the possible hindrance effect produced by helium atoms, and to ensure that the initial Cs${}_{2}$-C${}_{60}$ distance is larger than that at the intersection between the neutral and ionic electronic states, the size of the helium droplet was fixed to N=2090. The initial structure of the neutral, huge aggregate was built by combining an evolutionary algorithm [33] for N=300 and a geometric extension. In addition to classical calculations involving just one bead per particle (M=1), we allowed for an increasing level of quantization so that calculations for M=5 and 10 were also carried out.

Considering an initial Cs${}_{2}$-C${}_{60}$ velocity of only $v_{0}=25.5$ m/s, estimated from a Maxwell thermal distribution at T=2 K, we demonstrated that the redox reaction can not occur at thermal equilibrium. This finding is independent of the level of quantization, and can be explained by the fact that the intersection point between ionic and neutral potential energy curves is never reached. The heliophobic cesium dimer is unable to overcome the barrier of helium atoms and remains on the surface of the droplet creating a dimple, while the heliophilic fullerene is fully embedded inside.

At larger momenta for the beads associated with Cs atoms, raised by a factor of 4.5, the redox reaction becomes possible. The initial velocity then corresponds to a value of 129.1 m/s for M=1 (169.4 and 239.8 m/s for M=5 and 10, respectively). In this scenario, the heliophobic dimer can penetrate the droplet and performs a rotation from an initially T-shaped Cs${}_{2}$–C${}_{60}$ to a collinear arrangement upon reacting. The reaction probability grows with the level of quantization, going from 10% for M=1 up to almost certainty for M=10. By extrapolation of initial velocities to the case of M→∞, a value between 263 and 284 m/s is obtained. We note that these high velocities of the Cs${}_{2}$ reactant are needed only in case of an otherwise fully equilibrated droplet with a fullerene in a central position. Alternatively, the energy released upon Cs${}_{2}$ bond formation at the HND surface would produce an out-of-equilibrium situation in which either the Cs${}_{2}$ dimer would have enough kinetic energy to overcome the energetic barrier from the extrusion of helium atoms, rendering its approach to the fullerene molecule feasible, or the fullerene molecule could be excited from its center-of-the-droplet position towards the droplet surface and then react with the Cs${}_{2}$ dimer.

Finally, we note that vibrational excitations and impurity dynamics might be different in larger droplets. Therefore, as a future perspective, it would be interesting to carry out further classical calculations (M=1) for larger HNDs after renormalizing the He–He interaction potential to account for the fluid nature of the helium motion. This strategy is a simplified scheme to that proposed by Bonhommeau et al. [62], which was proven to be successful in a joint theory-experimental study of the He droplet-mediated aggregation and soft-landing deposition of metallic silver nanoparticles on carbon-based surfaces [63]. It appears sensible that the necessary initial velocity for submersion of the Cs${}_{2}$ dimer is lower using the renormalized He-He interaction potential, since the energy barrier related to the extrusion of He atoms would be smaller as well. In fact, the renormalized He–He potential would translate into a larger mean He-He distance and a softened He-He hard core, providing an enhanced dynamical fluxionality in the He–He motion to accommodate the intruding Cs${}_{2}$ dimer.

To summarize, our computational study confirms that the long-range electron-transfer or harpoon-type redox-reaction between Cs${}_{2}$ and C${}_{60}$, separated by helium due to their substantially different interaction with the droplet, involves a rotation of the Cs${}_{2}$ species at the cost of a substantial rearrangement of helium density. Consistent with the quantum nature of the helium fluid, this process becomes increasingly more probable as the quantization is better accounted for in the atomistic treatment. While confirming the reaction in principle, our study also indicates that higher relative Cs${}_{2}$-C${}_{60}$ velocities than those previously assumed [42] might be necessary to overcome the spatial hindrance. Therefore, an out-of-equilibrium situation appears most likely in the actual experimental scenario. It has also been pointed out that the energetic barrier for extrusion of He atoms, and then the initial velocity necessary for submersion of Cs${}_{2}$, should be smaller for the (50 times) larger HNDs produced in the experiment. Further classical PIMD (M=1) simulations for HNDs of experimentally determined size, using a renormalized He–He interaction potential accounting for the HNDs fluid nature [63], could provide insights into the HNDs size dependence of the initial velocity necessary for Cs${}_{2}$ submersion.

## Figures and Tables

**Table 1:** Parameters defining the analytical description of the Cs${}_{2}$-C${}_{60}$ and Cs${}_{2}^{+}$-C${}_{60}^{-}$ interactions at collinear (C) and T-shaped (T) arrangements, Equation (6), as well as the He-C${}_{60}$ interaction, Equation (4). Energies and distances are given in cm${}^{-1}$ and Å, respectively. These global units determine those corresponding to the different parameters. For instance, the unit of the $C_{6}$ coefficient parameter is cm${}^{-1}$Å${}^{6}$.

Cs${}_{2}$-C${}_{60}$			Cs${}_{2}^{+}$-C${}_{60}^{-}$			He-C${}_{60}$	
	C	T		C	T		
$A_{2}$	−2.22575d05	0	$B_{0}$	5.89589d03	4.52665d03		
$A_{4}$	9.66771d07	0	$B_{1}$	−1.37653d05	−1.03277d05		
$A_{6}$	−1.60255d10	9.598560d7	$B_{6}$	−3.91681d08	−3.39629d8	$F_{6}$	−1.90652d07
$A_{8}$	1.18367d12	−1.37271d11	$B_{12}$	6.40779d12	8.58655d12	$F_{8}$	3.76378d09
$A_{10}$	−4.10087d13	1.26854d13	$D_{0}$	5.14755d03	5.14755d03	$F_{10}$	−2.79654d11
$A_{12}$	5.48817d14	−4.37303d14	$D_{1}$	1.16141d05	1.16141d05	$F_{12}$	6.20350d12
$A_{14}$	0	5.43005d15	$q_{0}$,$q_{1}$	0	0		
$C_{6}$	−2.71882d08	−2.71882d08	$q_{6}$,$q_{12}$	−3.9	0		
$R^{\left(n\right)}$	12.5	12.5	$R^{\left(i\right)}$	28.0	20.0		

**Table 2:** Parameters defining analytical description of electron hopping probability at the collinear (C) and T-shaped (T) orientations, Equation (8). Units of *a* and *b* are m${}^{2}$/s${}^{2}$, while those of *c* and *d* are m${}^{4}$/s${}^{4}$.

	C	T
*a*	7047.479	1343.188
*b*	10,489.841	2226.989
*c*	469,477.985	13,407.221
*d*	2,797,586.355	43,037.030

## Data Availability

The data presented in this study are available in supplementary material.

## Supplementary Materials

The following are available online. Figures S1–S4: they present similar information as in Figure 5, Figure 6, Figure 7 and Figure 8 but obtained in the NVE ensemble, Video S1: movie showing how the redox reaction occurs, including the rotation of the Cs${}_{2}$ dimer before the charge transfer occurs as well as the evaporation of atomic helium.

## Sample Availability

Samples of the compounds are not available from the authors.
