# Supplementary material for: A Path Integral Molecular Dynamics Simulation of a Harpoon-Type Redox Reaction in a Helium Nanodroplet
Source: Molecules. 2021 Sep 24;26(19):5783. doi: 10.3390/molecules26195783 (PMC8510490; doi:10.3390/molecules26195783)
Supplement: Supplementary file 1 [file molecules-26-05783-s001.zip › Supplementary.pdf]

Supplementary Materials: A Path Integral Molecular Dynamics Simulation of a Harpoon-Type Redox Reaction in a Helium Nanodroplet

Alvaro Castillo-Garc [1,†] Andreas W. Hauser [2] María Pilar de Lara-Castells [1,*] and Pablo Villarreal [1,*]

1. Results obtained in the NVE ensemble in real time calculations

Calculations in the NVE micro-canonical ensemble have been also carried out to deliver results in real time and assess the conclusions presented in the main manuscript. Figures S1-S4 are the 'NVE counterparts' to Figures 5–8 of the main manuscript in NVT simulations.

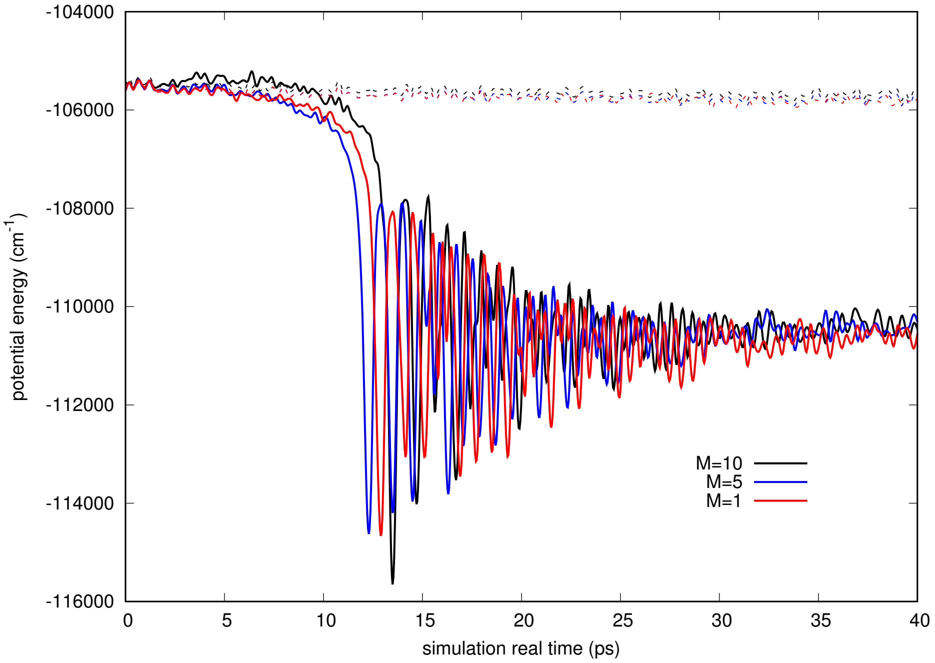

Figure S1. Similarly to Figure 5 of the main manuscript, the figure shows the time evolution of the potential energy of the Cs_2–C_{60}@He_{2090} system in the NVE ensemble, obtained in simulations performed at the indicated number of beads M. Solid lines correspond to initial relative velocities of the Cs_2–C_{60} interacting pair ($v_0 = 129.1$ m/s ($M = 1$, $v_0 = 169.4$ m/s ($M = 5$), $v_0 = 239.8$ m/s ($M = 10$). The dashed lines, maintaining the relation color/M, correspond to a common initial velocity v_0=25.4 m/s.

As in the NVT simulations (see Figure 5 of the main manuscript), it can be observed from Figure S1 that the reaction is not produced at thermal velocities. Only those trajectories obtained by applying a factor of 4.5 to the initial momenta become reactive. Independently of the number of beads, M, the potential minimum is reached in the interval $10-15$ ps. There is not a clear trend on the onset of reactivity. Apparently, the potential minimum is reached first for $M = 5$ while the opposite holds true for $M = 10$. The $M = 1$ case is intermediate.

The three panels of Figure S2 shows the evolution of relative Cs_2–C_{60} distances and velocities as a function of time for the common initial velocity $v_0 = 25.4$ m/s and $M = 1$ (lower panel), 5 (medium panel), and 10 (top panel). These NVE results completely agree with the NVT ones at T=2 K (see Figure 6 of the main manuscript). In particular, the crossing point between neutral/ionic potentials is never reached.

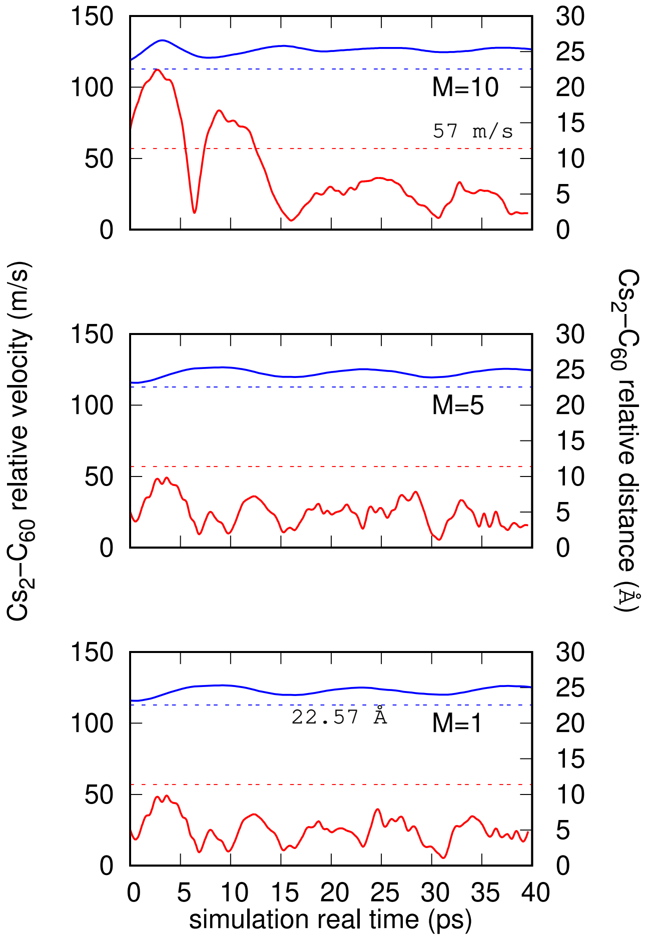

Figure S2. Similarly to Figure 6 of the main manuscript, the figure shows the evolution of non-reactive trajectories for the relative Cs_2–C_{60} distance (blue solid lines, right vertical axis) and velocity (red solid lines, left vertical axis) as a function of time. It has been obtained through PIMD simulations in the NVE emsemble. for the number M of beads considered, with the common initial relative velocity being $v_0 = 25.4$ m/s. Constant dashed lines indicate the critical Landau velocity (shown in red) as well as the crossing distance between neutral and ionic potential energy curves at the initial T-shaped orientation (shown in blue).

Similarly to Figure S2, the three panels of Figure S3 show the time evolution of relative Cs_2–C_{60} distances and velocities, considering the initial velocities $v_0 = 129.1$ m/s ($M = 1$, lower panel), 169.4 m/s ($M = 5$, medium panel), and 239.8 m/s ($M = 10$, top panel). There are clear similarities with the NVT calculations presented in the main manuscript (Figure 7), but in the NVE case the velocities at which the crossing neutral/ionic point is reached are clearly higher ($175 < v < 250$ m/s). As a result, the probability of hopping become lower: 10%, according to the Landau-Zener model (see Figure 3 of the main manuscript). Of course, this result is not conclusive as many more NVE trajectories should be launched to approach statistically converged probabilities, which is computationally unfeasible. Moreover, as an initial approach, the case of the Cs_2 dimer in linear orientation with respect to the C_{60} molecule should be also included as it favours the redox reaction (see Ref. 1).

As can be observed by comparing Figure S4 and Figure 8 from the main manuscript, there is again a remarkable similarity between NVT and NVE calculations. Yet, it is clear than the redox reaction is predicted to occur later under the NVE ensemble than using the NVT one. C_{60} remains close to its initial position, shown with a black solid line, implying a

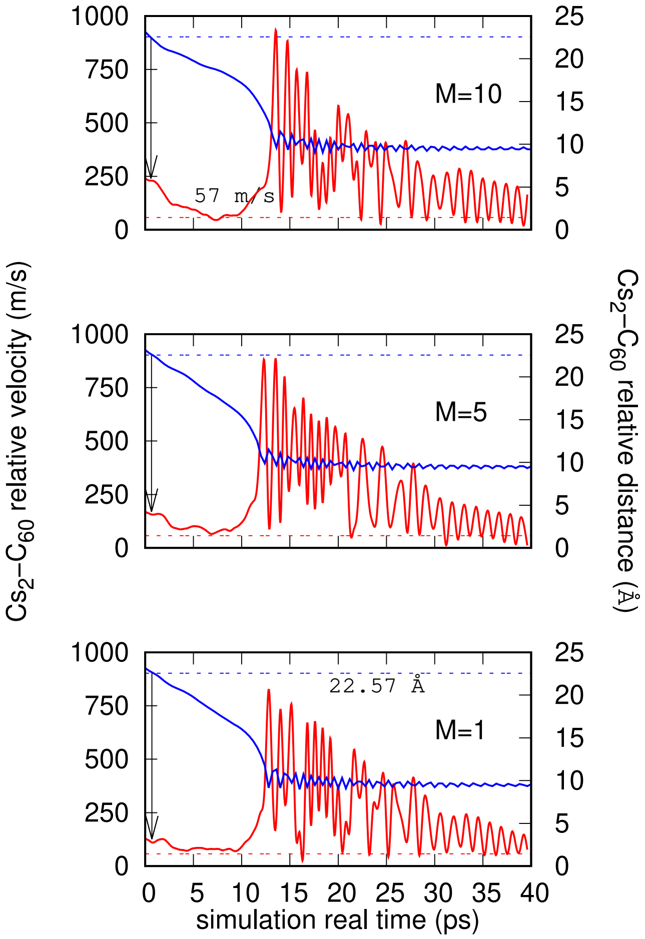

Figure S3. Similarly to Figure 7 of the main manuscript, the figure shows the evolution of reactive trajectories for relative Cs_2–C_{60} distance (blue solid lines) and velocity (red solid lines), plotted as a function of time, and obtained through simulations in the NVE ensemble for M beads ($M = 1, 5$, and 10) but with different initial relative velocities of $v_0 = 129$ m/s ($M = 1$), 169.4 m/s ($M = 5$), and 239.8 m/s ($M = 10$). The Landau critical velocity and the distance at the crossing between neutral and ionic potential energy curves at the T-shaped initial orientation are also shown with dashed red and blue lines, respectively. The arrows indicate the relative velocity of the reactants when they get the crossing distance between neutral and ionic potential energy curves.

small mobility. In addition, through the evolution of the maximum distance He–CM, we can observe that there is evaporation of atomic helium in the reactive case for $M = 10$. In stark contrast, no evaporation is observed for $M = 1$ and 5.

It can be observed from Figure S4 that the He–CM distance as a function of time acquires a pure linear behavior, pointing out that just one He atom escapes from the helium nanodroplet. By calculating the slope of the linear function, it is deduced that the He atom would escape with a kinetic energy of 2.5 cm^{-1}. Note that this value is is not far from the accepted value of $4-5$ cm^{-1} for evaporation in bulk helium. Interestingly, the probability of evaporation increases with the level of quantization, i.e. the M value. In fact, approaching the actual zero point enhances the floppiness of the aggregate. In spite that the evaporation is an important and usual effect for different reactions on He aggregates [2], the fraction of evaporated He atoms is very modest as reported in recent works: 25 in a cluster of 5000 He atoms in Ref. 3, and 6 out of 500 in Ref. 4.

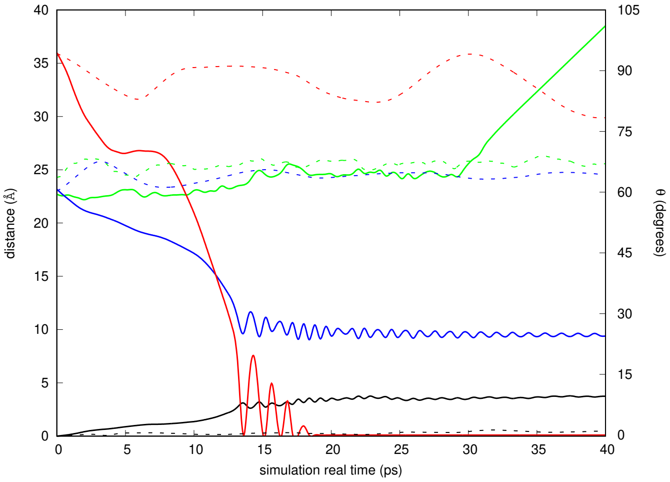

Figure S4. For M=10, evolution of relative Cs_2–C_{60} distance, *R* and orientation, θ, as function of time as obtained in NVE simulations. Dashed lines correspond to an initial velocity $v_0 = 25.4$ m/s, while solid lines show the simulation starting with $v_0 = 239.8$ m/s. Blue lines: relative distance between the two reactants; red lines: relative orientation; black lines: distance from C_{60} to its original position at time 0. It is similar to Figure 8 from the main manuscript but adding (green color, solid: reactive, dashed: non-reactive) the maximun distance of whatever be the He atom to the center of mass of the aggregate (referred to us He–CM).

References

1. de Lara-Castells, M.P.; Hauser, A.W.; Mitrushchenkov, A.O. Ab Initio Confirmation of a Harpoon-Type Electron Transfer in a Helium Droplet. *J. Phys. Chem. Lett.* **2017**, *8*, 4284.
2. Halberstadt, N.; Bonhommeau, D.A. Fragmentation dynamics of Ar_4He_{1000} upon electron impact ionization: Competition between ion ejection and trapping. *J. Chem. Phys.* **2020**, *152*, 234305. doi:10.1063/5.0009363.
3. Coppens, F.; Ancilotto, F.; Barranco, M.; Halberstadt, N.; Pi, M. Dynamics of impurity clustering in superfluid ^{4}He nanodroplets. *Phys. Chem. Chem. Phys.* **2019**, *21*, 17423.
4. Blancafort-Jorquera, M.; Vilà, A.; González, M. Quantum–classical approach to the reaction dynamics in a superfluid helium nanodroplet. The Ne_2 dimer and Ne–Ne adduct formation reaction Ne + Ne-doped nanodroplet. *Phys. Chem. Chem. Phys.* **2019**, *21*, 24218.
