# Supplementary figures and images for: A Path Integral Molecular Dynamics Simulation of a Harpoon-Type Redox Reaction in a Helium Nanodroplet
Source: Molecules. 2021 Sep 24;26(19):5783. doi: 10.3390/molecules26195783 (PMC8510490; doi:10.3390/molecules26195783)

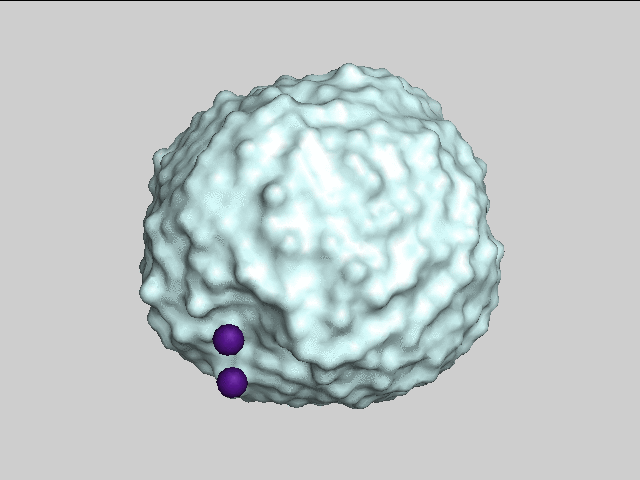

Supplement: Supplementary file 1 [file molecules-26-05783-s001.zip › Video S1.gif]
